# Bioinspired engineering of *Polygonum multiflorum* root nanovesicles regulates dermal papilla cells *in vitro* and stimulates human hair follicle growth *ex vivo*

**DOI:** 10.3389/fphys.2026.1808764

**Published:** 2026-04-30

**Authors:** Ramya Lakshmi Rajendran, Prakash Gangadaran, Mi Hee Kwack, Ji Min Oh, Chae Moon Hong, Young Kwan Sung, Byeong-Cheol Ahn

**Affiliations:** 1 BK21 FOUR KNU Convergence Educational Program of Biomedical Sciences for Creative Future Talents, Department of Biomedical Sciences, School of Medicine, Kyungpook National University, Daegu, Republic of Korea; 2 Department of Nuclear Medicine, School of Medicine, Kyungpook National University, Daegu, Republic of Korea; 3 Cardiovascular Research Institute, Kyungpook National University, Daegu, Republic of Korea; 4 Department of Immunology, School of Medicine, Kyungpook National University, Daegu, Republic of Korea; 5 Department of Nuclear Medicine, Kyungpook National University Hospital, Daegu, Republic of Korea

**Keywords:** dermal papilla cells, hair follicle regeneration, nanovesicles, polygonum multiflorum, β-catenin signaling

## Abstract

**Introduction:**

Polygonum multiflorum (PM), a traditional medicinal herb, is renowned for its regenerative effects on hair growth; however, its therapeutic application has been largely confined to crude extracts. Recent advances have highlighted plant-derived nanovesicles (PDNVs) as natural carriers of bioactive molecules. This study aimed to evaluate the hair growth–promoting potential of nanovesicles derived from the roots of Polygonum multiflorum (PM-NVs).

**Methods:**

PM-NVs were isolated from fresh PM roots using differential and density gradient ultracentrifugation. Their morphology was confirmed by transmission electron microscopy. Human dermal papilla cells (DPCs) were treated with PM-NVs to assess proliferation using the CCK-8 assay and β-catenin activation via Western blot and quantitative RT-PCR (qRT-PCR). *Ex vivo* cultured human hair follicles (HFs) were treated with PM-NVs to evaluate hair shaft elongation.

**Results:**

PM-NVs were spherical and efficiently internalized by DPCs. Treatment with PM-NVs significantly increased DPC proliferation in a dose-dependent manner, upregulated β-catenin protein levels, promoted its nuclear translocation, and enhanced the expression of downstream target genes (*Axin2, Lef1*, and *EP2*). *Ex vivo*, PM-NV treatment significantly enhanced human hair follicle elongation compared with controls, indicating stimulation of the hair growth phase.

**Conclusions:**

PM-NVs derived from Polygonum multiflorum promote dermal papilla cell (DPC) proliferation and activate β-catenin signaling, resulting in enhanced hair shaft elongation ex vivo. These findings suggest that PM-NVs represent a natural, biocompatible nanovesicle-based approach for hair follicle regeneration and hold potential as a therapeutic option for alopecia.

## Graphical Abstract

**Figure:**
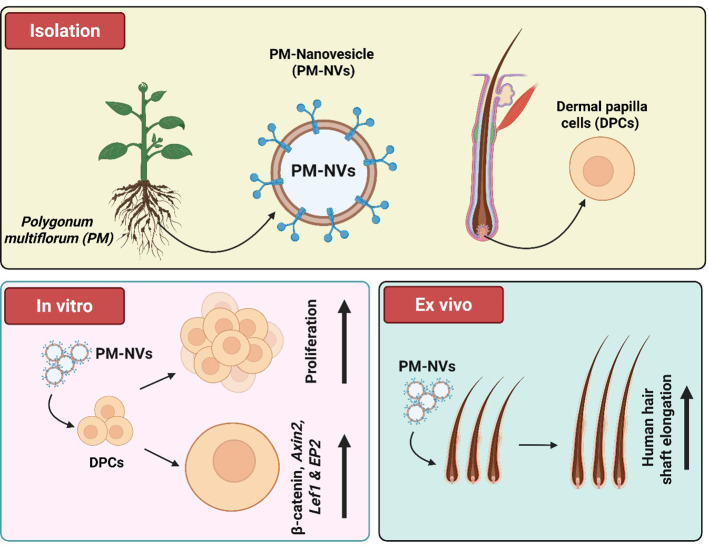

## 1 Background

Hair loss, also known as alopecia, encompasses various disorders characterized by partial or complete hair loss on the scalp or body (Devjani et al., 2023; Hamida et al., 2024). In the hair growth cycle, hair follicles (HFs) cyclically transition through three main phases: anagen (growth), catagen (regression), and telogen (resting). Each stage is characterized by distinct morphological, functional, and molecular alterations within HFs (Lin et al., 2022). Among the various cell types in HFs, dermal papilla cells (DPCs) act as key regulators of HF maintenance and regeneration (Zhang et al., 2024).

*Polygonum multiflorum* (PM), a flowering plant belonging to the buckwheat family, is a popular perennial herb known for its regenerative properties. It is known as *He Shou Wu* in traditional Chinese medicine (Bounda and Feng, 2015). Several reports have demonstrated that PM extract possesses hair regenerative, proangiogenic, and antiapoptotic properties. Notably, all these studies used extracts primarily derived from dried PM roots (Chen et al., 2011; Li et al., 2015; Chen et al., 2018; Chen T-Y. et al., 2024). Plant-derived nanovesicles (PDNVs) have recently attracted attention as natural, biocompatible delivery vehicles in the field of regenerative medicine (Zheng et al., 2025). Similar to mammalian extracellular vesicles, nanovesicles are naturally optimized delivery vehicles that carry functional genetic material, proteins, and lipids for inducing cellular responses. PDNVs exhibit promising therapeutic activities, such as anti-inflammatory, anti-tumor, and wound-healing effects (Nemati et al., 2022; Xu et al., 2023). In contrast, plant extracts are nonspecific and may degrade quickly or cause off-target effects (Zeng et al., 2024).

Although the hair growth-promoting effects of PM have been demonstrated using extracts, the effects of PM-derived nanovesicles (PM-NVs) on DPC activation and hair regrowth remain unexplored. In this current study, we used fresh PM roots to generate PM-NVs and assessed their effects on the activation of DPCs and the promotion of HF elongation.

## 2 Experimental design

### 2.1 DPC culture

HFs in Anagen-phase were collected from scalp skin after taking informed consent from the patients. This study was approved by the Medical Ethics Committee of Kyungpook National University Hospital (Daegu, Republic of Korea) and was conducted in accordance with the principles and guidelines of the Declaration of Helsinki (IRB No: KNU-2022-0497).

HFs bulbs of dissected to isolated the DPCs, cells were moved to tissue culture plates coated with bovine type I collagen, and cultured in low-glucose DMEM (HyClone, Logan, UT, USA) supplemented with 20% heat-inactivated FBS, 1% antibiotic–antimycotic and 1 ng/mL bovine fibroblast growth factor (Sigma, MO, USA) at 37 °C. The explants were maintained for 7 days, with medium changes performed every 3 days. After isolation, DPCs were plated onto 100-mm culture dishes with low-glucose DMEM supplemented with 10% heat-inactivated FBS. The DPCs were passaged based on to the confluence %, and second-passage cells were used in this study (Rajendran et al., 2017).

### 2.2 Generation, isolation, and purification of nanovesicles

In total, 50 g of PM roots were washed with distilled water to remove the soil, sliced into smaller pieces, and blended with PBS in a commercially available mini blender until they were well blended. The blended root juice was filtered with a mesh filter (200–300 µm), transferred to 50 mL tubes, and centrifuged sequentially at 4, 000 ×g for 1 h and then at 10, 500 ×g for 1 h. The supernatants were moved to ultracentrifuge tubes. Subsequently, the samples were ultracentrifuged in Optima™ L-100 XP ultracentrifuge (Beckman Coulter, Brea, CA, USA) at 100, 000 ×g for 1 h. Then, discarded the supernatant, and the pellets were reconstituted with PBS. The PM-NVs (pellets) were purified using two-step OptiPrep™ Density Gradient Medium (iodixanol) (Sigma-Aldrich, St. Louis, MO, USA) density gradient ultracentrifugation at 120, 000 ×g for 3 h. The 20% and 60% iodixanol solutions were prepared by diluting the stock OptiPrep™ (60% iodixanol) solution with sterile PBS according to the manufacturer’s instructions. The final PM-NVs were gathered from the between 20% and 60% iodixanol layers’ interface, the samples were carefully collected from the top of the gradient using a micropipette and transferred to sterile microcentrifuge tubes and stored at −80 °C. The protein concentration of PM-NVs was quantified using a bicinchoninic acid (BCA) protein assay, a widely used method for determining total protein in plant extracellular vesicle preparations (Ju et al., 2013; Zhang et al., 2016; Cui et al., 2024).

### 2.3 TEM

The PM-NV pellets were resuspended in 2% paraformaldehyde (100 µL). Then, PM-NV (16.62 µg) suspension was placed on top of Formvar/carbon-coated EM grids and protected with aluminum foil for 20 min to prevent drying. For washing, PBS (100 µL) was placed onto parafilm, and the grids were transferred using sterile forceps. The PM-NVs containing grids were then incubated with 1% glutaraldehyde (50 µL)at 25 °C–30 °C for 5 min and washed gently with distilled water for 2 min. PM-NVs were negatively stained with 2% uranyl acetate for 5 min and followed by seven washes with distilled water (2 minutes each). Finally, the grids were air-dried and visualized under an HT 7700 transmission electron microscope (Hitachi, Tokyo, Japan) (Gopal et al., 2024).

### 2.4 Internalization assay

PM-NVs were labeled with the lipophilic dye 1, 1’--dioctadecyl-3, 3, 3’, 3’-tetramethylindodicarbocyanine (DiD; Thermo Fisher Scientific, Waltham, MA, USA) for 20 min, room temperature (RT, 20–25 °C) and purified to remove free dye. DPCs were incubated with DiD-PM-NVs (2.66 µg/mL) for 4 h at 37 °C, washed, and counterstained with Vectashield mounting medium with DAPI (Vector Laboratories, Burlingame, CA, USA) to visualize nuclei. Images were acquired using a AXIO fluorescence microscope (Zeiss, Baden-Württemberg, Germany).

### 2.5 CCK-8 assay

DPCs (5 × 10³ cells/well) were seeded overnight into 96-well plates and then incubated with varying concentrations of PM-NVs (0, 0.66, 1.33, 1.99 and 2.66 µg/mL) for 24 h at 37 °C in a CO_2_ incubator. A 10 µL aliquot of CCK-8 solution (Dojindo Molecular Technologies, Kyushu, Japan) was added to each well and incubated for 2 h, after which absorbance at 450 nm was recorded using a microplate reader to assess cell proliferation, as recommended by the manufacturer.

### 2.6 Treatment of DPCs with PM-NVs for western blotting and qRT-PCR

DPCs were incubated with or without PM-NVs (2.66 µg/mL) for 24 h at 37 °C in a CO_2_ incubator. Then, the cells were harvested for western blotting and qRT-PCR.

### 2.7 Western blotting

Western blot analysis was performed as defined previously (Gopal et al., 2024). Whole-cell lysate was prepared in RIPA buffer (Sigma, MO, USA). The same amounts of protein were loaded and separated using 10% SDS–PAGE. Proteins were electrotransferred onto polyvinylidene difluoride (PVDF) membranes (Millipore, MA, USA). They were initially probed with primary β-catenin (1:5, 000, Cell Signaling Technology (CST), USA) or β-actin (1:10, 000, CST, USA) antibody and following primary antibody incubation, membranes were treated with an HRP-conjugated secondary antibody (1:10, 000; CST, USA). The signals were detected using enhanced chemiluminescence (GE Healthcare, IL, USA), according to the manufacturer’s instructions. Images of the blots were cropped and formatted using PowerPoint (Microsoft, WA, USA). Contrast was modified, if required, to improve image clarity, and band intensities were analyzed with GelQuant.NET (BiochemLabSolutions.com, CA, USA).

### 2.8 Immunofluorescence assay

DPCs were seeded at a density of 1 × 10^4^ cells per well in a 4-well chamber slide and cultured under standard conditions as mentioned above overnight. Cells were treated with vehicle control (DMSO), PM-NVs; 2.66 µg/mL, or PM-NVs in combination with the β-catenin pathway inhibitor XAV939 (10 µM; Sigma, MI, USA) for 24 h. Following treatment, cells were washed three time with PBS and fixed with 4% paraformaldehyde for 10 min at room temperature. Cells were then permeabilized with 0.1% Triton X-100 for 10 min and blocked with 3% bovine serum albumin (BSA; Sigma, MI, USA) for 1 h. to minimize nonspecific binding. Subsequently, cells were incubated overnight at 4 °C with a primary antibody against anti-rabbit β-catenin (1:200 dilution; CST, USA). After washing with PBS three times, cells were incubated with Goat anti-Rabbit IgG (H+L) Highly Cross-Adsorbed Secondary Antibody, Alexa Fluor™ Plus 488 secondary antibody (1:200; Invitrogen, California, USA) for 1 h at room temperature in the dark. washed, and counterstained with Vectashield mounting medium with DAPI (Vector Laboratories, Burlingame, CA, USA) to visualize nuclei. Images were acquired using a AXIO fluorescence microscope (Zeiss, Baden-Württemberg, Germany).

### 2.9 qRT-PCR

TRI Reagent (Bioscience Technology, South Korea) used to isolate the RNA and then, cDNA was synthesized using the High-Capacity cDNA Synthesis Kit (Applied Biosystems, CA, USA), following the manufacturer’s instructions. RT-PCR was ran as described previously (Rajendran et al., 2021). Each reaction mixture consisted of SsoAdvanced™ Universal SYBR Green Supermix (Bio-Rad Laboratories, CA, USA), 50 ng of cDNA template, and 10 μM primers. The primer sequences used for mRNA analysis were as follows: Axin2 forward (5′→3′) AGCTAGGAGTGCGTTCATGGTT and reverse (5′→3′) GGAGGGACGTAGTGCAAAGC; Lef1 forward (5′→3′) CAGGAGCCCTACCACGACAA and reverse (5′→3′) CCTCCATCTGGATGCTTTCC; EP2 forward (5′→3′) GGAGGGCGCATCTCTTTTC and reverse (5′→3′) GGAGTCATTGGAGGCATTGC; and 18S rRNA forward (5′→3′) CGGCTACCACATCCAAGGAA and reverse (5′→3′) GCTGGAATTACCGCGGCT. Amplification was run in a CFX96 touch real-time PCR system (Bio-Rad) using the following PCR cycling parameters: PCR reactions were performed with an initial denaturation at 95 °C for 10 min, followed by 40 cycles of 95 °C for 15 s and 60 °C for 1 min. Differences between samples and controls were calculated using real-time PCR analysis software (Bio-Rad).

### 2.10 Human hair shaft elongation assay

Human HFs were obtained and cultured according to a previously reported protocol (Kwack et al., 2017). Biopsy samples were collected from the occipital scalp of male patients with androgenic alopecia undergoing hair transplantation. Written informed consent was obtained from the patients. The Medical Ethics Committee of Kyungpook National University Hospital (Daegu, Korea) approved all experimental procedures (IRB No: KNU-2022-0497). Nonbalding scalp HFs were isolated, and the subcutaneous fat region, including the lower follicular portions, was separated from the epidermal and dermal layers. The HFs were microscopically isolated under a binocular microscope using forceps and maintained in Williams’ E medium without phenol red (Sigma, MI, USA) at 37 °C in a humidified atmosphere under 5% CO_2_. Subsequently, HFs were treated with PBS (control) or PM-NVs (0, 1.66, 3.32 and 6.65 µg/mL). Hair shaft elongation was then imaged and measured on day 6.

### 2.11 Statistical analysis

Data are expressed as mean ± standard deviation (SD). Differences between pairs of groups were statistically analyzed using Student’s t-test in Excel (Microsoft) or GraphPad Prism version 10.5.0 (774) (GraphPad Software Inc., San Diego, CA, USA). p < 0.05 was considered statistically significant.

## 3 Results and discussion

### 3.1 Isolation and characterization of PM-NVs

**Figure 1:**
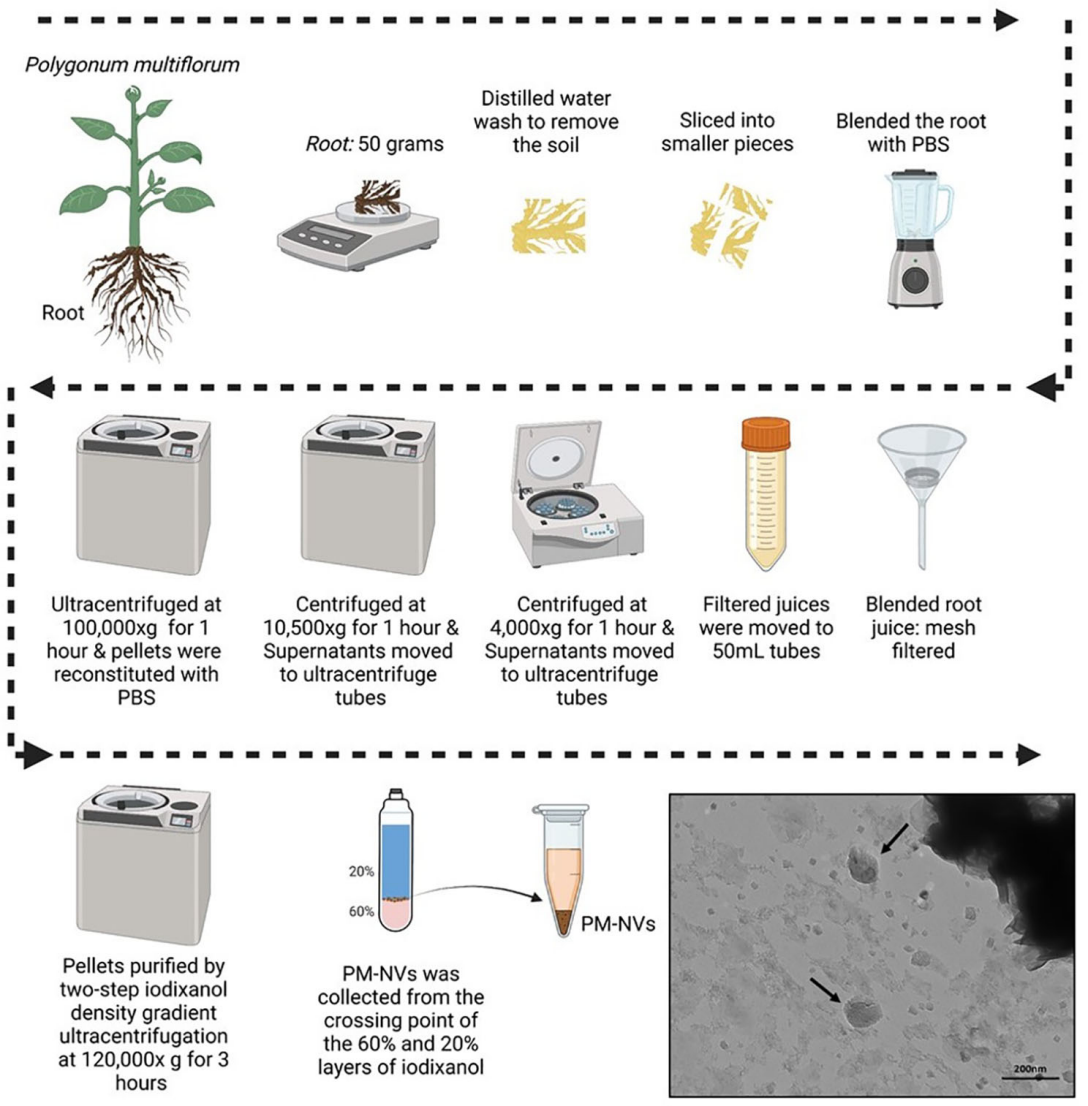
Isolation and purification of *Polygonum multiflorum*-derived nanovesicles (PM-NVs). Schematic illustration of the preparation of PM-NVs from *Polygonum multiflorum* roots. TEM image confirms the vesicle morphology. The black arrow indicates sphere-like PM-NVs (scale bar = 200 nm).

PM-NVs were successfully isolated from root samples using a multistep differential ultracentrifugation process. Further purification was performed using iodixanol gradient ultracentrifugation. This enabled the collection of a distinct nanovesicle layer at the interface between 20% and 60% iodixanol (**Figure 1**). TEM confirmed the presence of sphere-like structures consistent with extracellular vesicles/nanovesicles (Cho et al., 2022; Chen X. et al., 2024), indicating the successful isolation of PM-NVs exhibiting typical nanovesicular morphology and integrity.

### 3.2 Internalization of PM-NVs leads to increased proliferation and activation of the Β-catenin signaling pathway in DPCs

**Figure 2:**
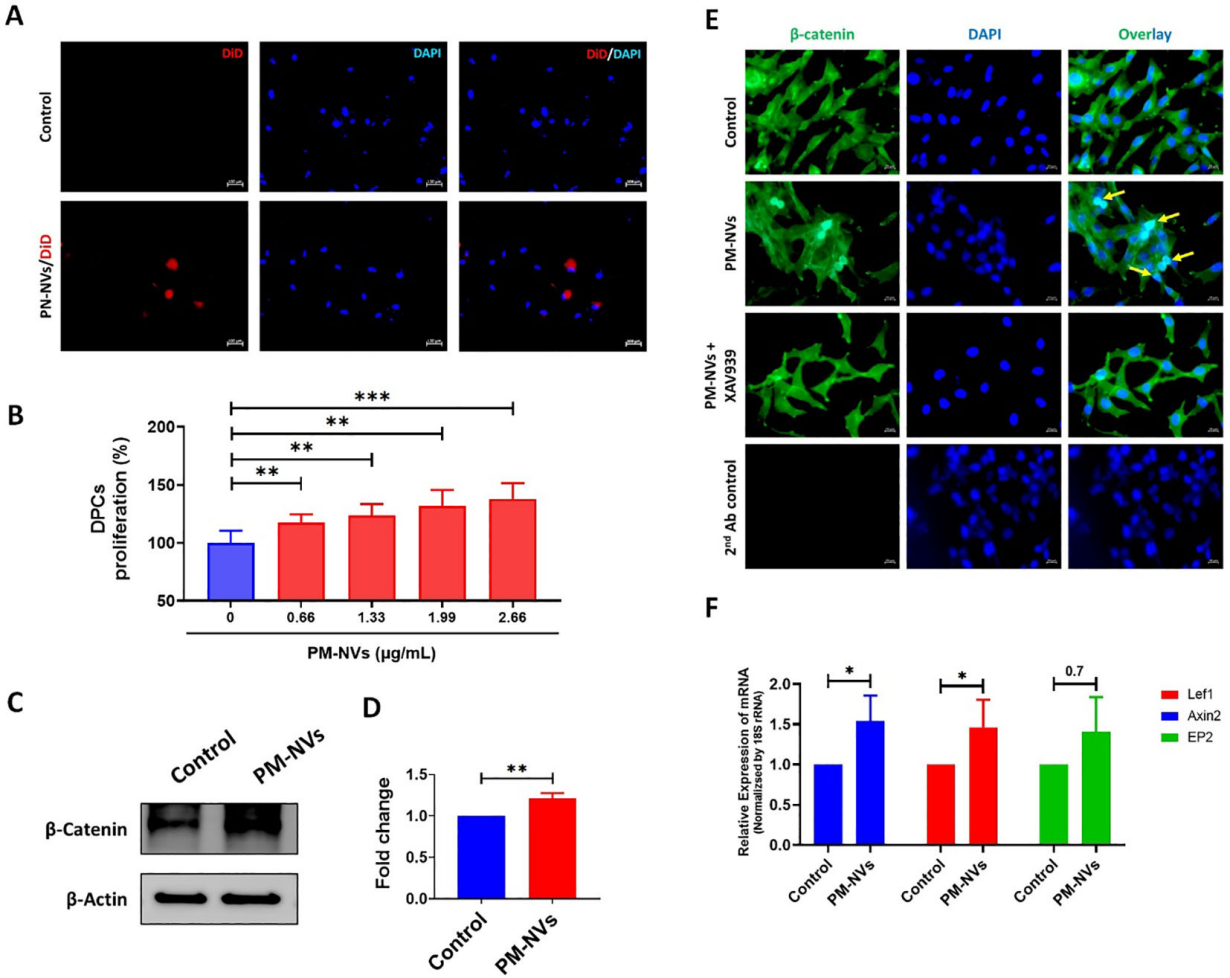
Internalization, proliferation, and signaling of DPCs treated with PM-NVs. **(A)** Fluorescent microscopy images showing the internalization of DiD-labeled PM-NVs (red) into DAPI-stained nuclei of DPCs (blue) (Scale bar: 100µM). **(B)** Quantification of DPC proliferation after 24 h post-treatment with PM-NVs (0.66, 1.33, 1.99 and 2.66 µg/mL) (n = 5). **(C)** Western blot analysis of β-catenin and β-actin (loading control) expression after 24 h in DPCs treated with PM-NVs (2.66 µg/mL). **(D)** Densitometric analysis of β-catenin levels normalized to β-actin (n = 3). **(E)** Fluorescent microscopy images showing the translation of β-catenin (green) expression after 24 h in DPCs treated with, control (DMSO), PM-NVs (2.66 µg/mL) with or without XAV939 (10µM); DAPI-stained nuclei of DPCs (blue) (Scale bar: 20µM). **(F)** RT-qPCR showing the relative expression levels of *Axin2, Lef1*, and *EP2* in 2.66 µg/mL of PM-NV-treated cells (normalized to *18S rRNA* levels)[control (n = 3) & PM-NVs (n=6)]. Data are expressed as the mean ± SD. Data were analyzed using Student’s t-test. *p < 0.05, **p < 0.01 and ***p < 0.001.

To assess cellular internalization, DiD-labeled PM-NVs were incubated with DPCs. Fluorescent microscopy merged images revealed clear internalization of DiD-labeled PM-NVs within DPCs, around DAPI-stained nuclei (blue); however, this was not noted in the control group (**Figure 2A**), confirming that PM-NVs can be efficiently taken up by DPCs. The effects of PM-NVs on DPC proliferation were assessed using increasing concentrations of PM-NVs (0.66, 1.33, 1.99 and 2.66 µg/mL). A dose-dependent increase in DPC proliferation was observed, with significant enhancement noted at all concentrations relative to the control (**Figure 2B**). Notably, compared to the control, the proliferation rate was significantly higher at 2.66 µg/mL of PM-NVs (p < 0.001). This finding indicates a potential therapeutic effect on DPCs, in agreement with a previous report that exosome-like nanovesicles or exosomes extracted from *Withania somnifera* (commonly known as ashwagandha) could promote DPC proliferation (Dell’acqua et al., 2024). The Wnt/β-catenin signaling pathway is a crucial regulator of hair follicle regeneration and cycling. Activation of Wnt ligands stabilizes cytoplasmic β-catenin, enabling its translocation into the nucleus, where it interacts with TCF/LEF transcription factors. This interaction induces the expression of genes involved in dermal papilla cell proliferation and hair follicle development (Choi, 2020; Liu et al., 2022). The molecular mechanism underlying the observed proliferative effect was explored by examining the expression of β-catenin, a key regulator of hair growth (Bejaoui et al., 2019; Yan et al., 2024). Western blot analysis revealed that the level of β-catenin protein was higher (p < 0.01) in PM-NV-treated DPCs than in the control (**Figures 2C, D**) and PM-NVs treatment markedly promoted β-catenin nuclear translocation in DPCs compared to control, this effect was attenuated by XAV939, confirming Wnt/β-catenin pathway involvement (**Figure 2E**). In a previous study, PM extract was found to upregulate β-catenin expression for promoting hair growth (Park et al., 2011). Similar to our PM-NVs, garlic-derived exosomes could stimulate hair growth by activating the Wnt/β-catenin signaling pathway (Inan Yuksel et al., 2023). Moreover, RT-qPCR revealed that PM-NV treatment increased the mRNA expression levels of canonical Wnt target genes (*Axin2*, *Lef1*, and *EP2*) *(*Kwack et al., 2011; Liu et al., 2022), with *Axin2 (p<0.05), Lef1(p<0.05)* and *EP2* (p=0.07) showing the upregulation compared to control (**Figure 2F**). In particular, two active compounds identified in the PM extract namely, 2, 3, 5, 4′-tetrahydroxystilbene-2-O-*β*-D-glucoside (TSG) and emodin have been reported to possess hair-growth-promoting properties (Yon et al., 2016; Xu et al., 2017). Considering that PM-NVs are derived from the same plant source, it is possible that these PM-NVs may also encapsulate or be enriched with such bioactive compounds like TSG and emodin, thereby contributing to their observed hair-regenerative effects. However, additional studies are required to confirm the presence of these compounds within PM-NVs. These results suggest that PM-NVs activate the β-catenin signaling pathway, potentially enhancing the proliferation of DPCs.

### 3.3 PM-NVs promote hair shaft elongation in *ex vivo-*cultured human HFs

**Figure 3:**
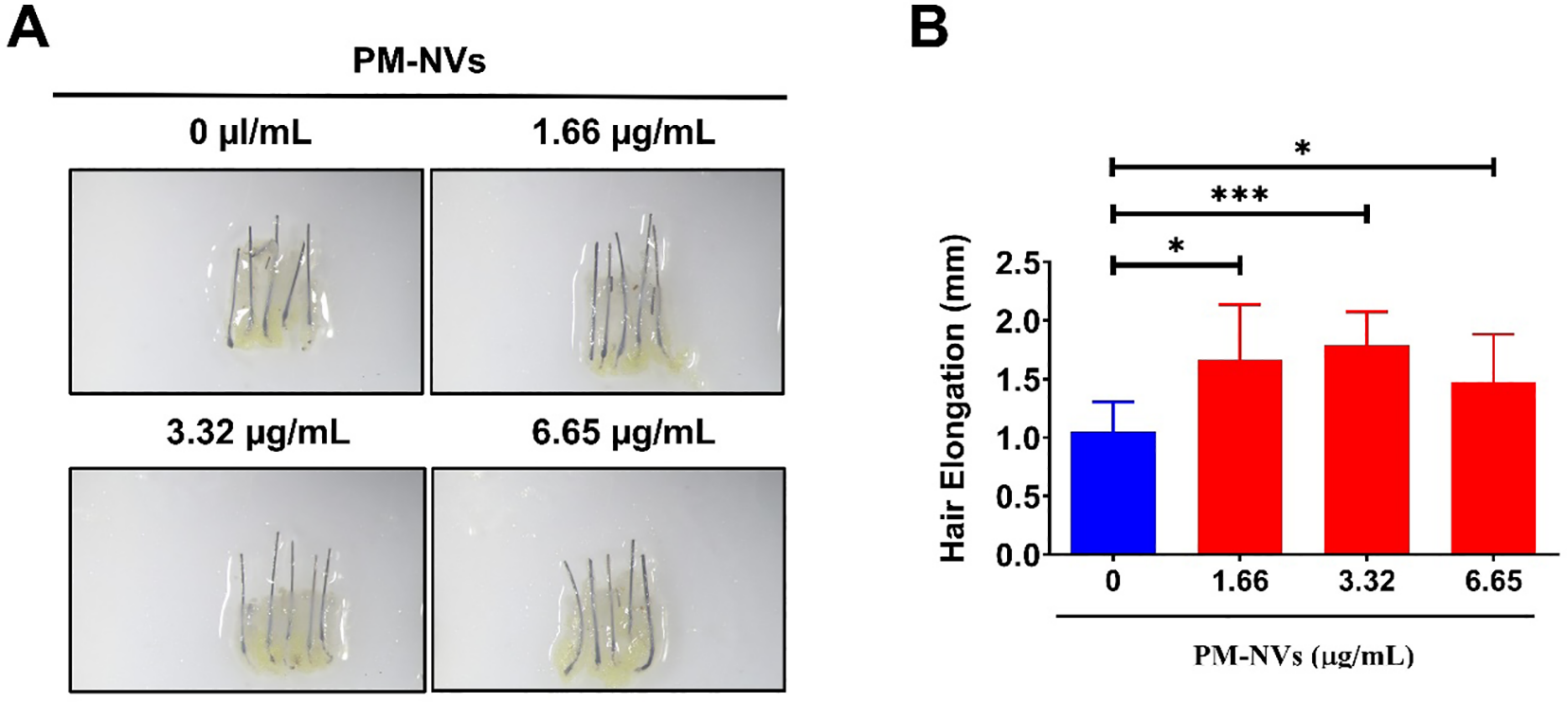
Effect of PM-NVs on human hair shaft elongation *ex vivo*. **(A)** Macroscopic images of human hair follicles treated with different concentrations of PM-NVs (0, 1.66, 3.32 and 6.65 µg/mL). **(B)** Quantification of hair elongation after 6 days of treatment (n = 5) with PM-NVs. Data are presented as the mean ± SD. Data were analyzed using Student’s t-test. *p < 0.05 and ***p < 0.001.

To examine the therapeutic effects of PM-NVs for promoting hair growth, human HFs were cultured *ex vivo* and treated with various concentrations of PM-NVs (0.66, 1.33, 1.99 and 2.66 µg/mL). Macroscopic images of HFs revealed visibly greater hair shaft length in the PM-NV-treated groups than in the control (**Figure 3A**). Quantitative analysis revealed significantly greater HF elongation on treatment with 1.66and 3.32 µg/mL of PM-NVs; the mean length was more than 1.5 mm in the PM-NV-treated groups compared to approximately 1.0 mm in the control group (**Figure 3B**, p < 0.001 and p < 0.05). In a previous study, PM extract was found to promote the telogen to anagen transition (Shin et al., 2020). This suggests that PM-NVs not only stimulate DPC proliferation but also promote hair shaft growth.

## 4 Conclusions

In this study, PM-NVs were successfully isolated from PM roots, and their morphology and uptake by DPCs were assessed. PM-NVs promoted DPC proliferation, activated β-catenin signaling, and upregulated β-catenin target genes. *Ex vivo*, they enhanced hair shaft elongation, suggesting their potential for hair regeneration. Unlike drugs, PM-NVs represent a naturally derived, biocompatible, and potentially safer alternative for therapeutic use.

Future studies should focus on identifying the precise molecular cargo of PM-NVs, such as RNA, proteins, or small metabolites responsible for their bioactivity; exploring their effects in *in vivo* animal models of hair loss to determine their safety, biodistribution, and regenerative efficacy; developing scalable production and purification techniques; and assessing their therapeutic applicability for other skin-related diseases.

## Data Availability

The original contributions presented in the study are included in the article/supplementary material. Further inquiries can be directed to the corresponding author.
